# Digital whole-community phenotyping: tracking morphological and physiological responses of plant communities to environmental changes in the field

**DOI:** 10.3389/fpls.2023.1141554

**Published:** 2023-05-09

**Authors:** Vincent Zieschank, Robert R. Junker

**Affiliations:** ^1^ Evolutionary Ecology of Plants, Department of Biology, Philipps-University Marburg, Marburg, Germany; ^2^ Department of Environment and Biodiversity, University of Salzburg, Salzburg, Austria

**Keywords:** digital phenotyping, grassland, land-use, morphology, physiology, plant community, PlantEye F500, trait-based ecology

## Abstract

Plant traits are informative for ecosystem functions and processes and help to derive general rules and predictions about responses to environmental gradients, global change and perturbations. Ecological field studies often use ‘low-throughput’ methods to assess plant phenotypes and integrate species-specific traits to community-wide indices. In contrast, agricultural greenhouse or lab-based studies often employ ‘high-throughput phenotyping’ to assess plant individuals tracking their growth or fertilizer and water demand. In ecological field studies, remote sensing makes use of freely movable devices like satellites or unmanned aerial vehicles (UAVs) which provide large-scale spatial and temporal data. Adopting such methods for community ecology on a smaller scale may provide novel insights on the phenotypic properties of plant communities and fill the gap between traditional field measurements and airborne remote sensing. However, the trade-off between spatial resolution, temporal resolution and scope of the respective study requires highly specific setups so that the measurements fit the scientific question. We introduce small-scale, high-resolution digital automated phenotyping as a novel source of quantitative trait data in ecological field studies that provides complementary multi-faceted data of plant communities. We customized an automated plant phenotyping system for its mobile application in the field for ‘digital whole-community phenotyping’ (DWCP), capturing the 3-dimensional structure and multispectral information of plant communities. We demonstrated the potential of DWCP by recording plant community responses to experimental land-use treatments over two years. DWCP captured changes in morphological and physiological community properties in response to mowing and fertilizer treatments and thus reliably informed about changes in land-use. In contrast, manually measured community-weighted mean traits and species composition remained largely unaffected and were not informative about these treatments. DWCP proved to be an efficient method for characterizing plant communities, complements other methods in trait-based ecology, provides indicators of ecosystem states, and may help to forecast tipping points in plant communities often associated with irreversible changes in ecosystems.

## Introduction

Trait-based ecology has emerged as a tool to derive general rules and predictions about functional changes in communities in response to environmental gradients, global change components and other perturbations such as species loss or invasions (Lavorel and Garnier, 2002; Funk et al., 2017; de Bello et al., 2021). Quantitative measurements of traits complementing species inventories thus provide the linkage to ecosystem functions and processes (Díaz et al., 2007; Laliberté and Legendre, 2010; Cadotte et al., 2013). For instance, the distribution and variability of traits in grassland communities was related to primary productivity (Mason and de Bello, 2013; Turnbull et al., 2013; Liu et al., 2015), ecosystem stability (Turnbull et al., 2013), and the diversity of organisms in higher trophic levels that interact with the plants (Junker et al., 2015). One major advantage of trait-based assessments of plant diversity is the transferability and thus generalization of findings to other plant communities that may be composed of a different set of species: communities experiencing the same environmental conditions have been shown to converge in species traits despite differences in taxonomic composition (Fukami et al., 2005), indicating that functional traits are of key importance in community ecology and reveal mechanisms complementary to taxonomical information in community assembly (HilleRisLambers et al., 2012; Meiners et al., 2015).

Direct field measurements of plant traits using caliper rules, scales, office scanners, and handheld spectrometers is the traditional and maybe most common and direct way of assessing the functional composition and diversity of local plant communities (Pérez-Harguindeguy et al., 2016; Kuppler et al., 2017; Junker et al., 2019; de Bello et al., 2021; Escudero et al., 2021). These measurements are specific to the location and the phenotyped species and precisely measure selected traits of individual plants. As these approaches are associated with a high workload, feasible sampling schemes often cannot include all species present in a community (de Bello et al., 2021). In recent years, data bases such as the Plant Trait Database TRY (Kattge et al., 2020) have been established to enable future studies that are built on a broader data foundation or can cover much larger scopes. These data bases led to unprecedented insights into the ecology and evolution of plants across taxonomic boundaries (Diaz et al., 2016) and to numerous macroecological studies revealing plant responses to large-scale biotic or abiotic gradients (e.g. Kuppler et al., 2020). While theoretically providing individual trait measurements supplemented by information on local adaptations and intraspecific variability, in most cases the data extracted from TRY is used at the level of species mean trait values.

Whereas manual, i.e. ‘low-throughput’, phenotyping methods dominate ecological field studies, in agricultural research and applications ‘high-throughput phenotyping’ became standard (Dhondt et al., 2013; Rosenqvist et al., 2019). A variety of approaches are available to acquire phenotypic raw data such as cameras for RGB color recording, multispectral units for reflectance measuring in the visible, infrared, or near-infrared spectrum, lasers that measure distance for 3D imaging and thermal sensors, among others (White et al., 2012; Barbedo, 2019). The application of such devices is often limited because either individual plants need to be transported to a stationary scanner (plant-to-sensor) or scanners are installed in greenhouses or outdoor facilities where they move over a defined set of plants (sensor-to-plant) (Busemeyer et al., 2013; Fiorani and Schurr, 2013; Demidchik et al., 2020). Thus, for an application in field-based ecology plant scanners are required that are mobile and therefore applicable under field conditions, independent of a stationary infrastructure.

In ecological field studies, remote sensing makes use of freely movable devices like satellites or unmanned aerial vehicles (UAVs). They provide large-scale spatial and temporal data at a resolution that allows for the classification of land-cover types or the prediction of different aspects of diversity (Zhu and Woodcock, 2014; Wachendorf et al., 2018; Cavender-Bares et al., 2020; Sun et al., 2021). Additionally, they are used in ecosystem monitoring to create habitat maps or capture characteristics of plant communities (Cruzan et al., 2016; Blackburn et al., 2021). At present there are many different types of sensors to be used with satellites and UAVs to record different aspects of plant diversity, cover, and status (Barbedo, 2019), and the possibilities for three-dimensional measurements also increased in recent years (Lepczyk et al., 2021). Adopting such methods for community ecology on a smaller scale may provide novel insights and a more complete view on the phenotypic properties of plant communities and may fill the gap between traditional field measurements and airborne remote sensing. Newly emerged ground-based phenomics platforms like ‘Phenomobile’ or ‘FieldExplorer’ (plantphenomics.org.au) for plant trait phenotyping are most commonly used by crop scientists to record growth and physiological traits of major crops and link them to specific genotypes (Qiu et al., 2019). While those might also be adaptable for ecological field studies, the trade-off between spatial resolution, temporal resolution and scope of the respective study requires highly specific setups so that the measurements fit the scientific question (Gehan and Kellogg, 2017; D'Urban Jackson et al., 2020).

We introduce small-scale, high-resolution digital automated phenotyping of plant communities as a novel source of quantitative trait data in ecological field studies that provides complementary multi-faceted data while reducing the workload compared to classic trait measurements. In this study we used an automated plant phenotyping system (PlantEye F500, Phenospex, Heerlen, The Netherlands) to collect high-resolution multispectral information on plant communities in the field while simultaneously also capturing the 3-dimensional structure of the vegetation. This method will be referred to as ‘digital whole-community phenotyping’ (DWCP) in the following.

We customized a MicroScan device (Phenospex, Heerlen, The Netherlands) equipped with the scanner PlantEye F500 in such way that it is fully mobile, can be mounted in the field without limitations, and runs on a mobile battery. This system generates 3D point clouds complemented with multispectral information, and allowed us to track short-term responses of plant communities to experimental land-use changes in a common garden. The common garden contained grass sods originating from three regions of the Biodiversity Exploratories, a long-term research platform in Germany to study the effects of land use on biodiversity and ecosystem processes (Fischer et al., 2010). In the common garden in Marburg, we subjected each of the sods to one of four land-use treatments of differing intensity. We scanned each sod of the common garden multiple times over two growing seasons (*n* = 11 sampling events) recording a time-series of morphological and physiological changes in the plant communities. We extracted a total of 14 parameters from each scan: nine morphological parameters derived from the 3D point cloud and five physiological variables derived from multispectral information.

To compare the results of DWCP with classical methods, we additionally manually measured quantitative vegetative traits of plant species on all sods. Using random forest analysis and data from digital and manual phenotyping as well as vegetation analyses, we classified each sod in each sampling event to its provenance and its experimental land-use treatment. Our results demonstrate the potential of automated plant phenotyping systems in assessing morphological and physiological traits and responses to environmental factors of whole plant communities in the field.

## Methods

### Digital whole-community phenotyping

**Figure 1:**
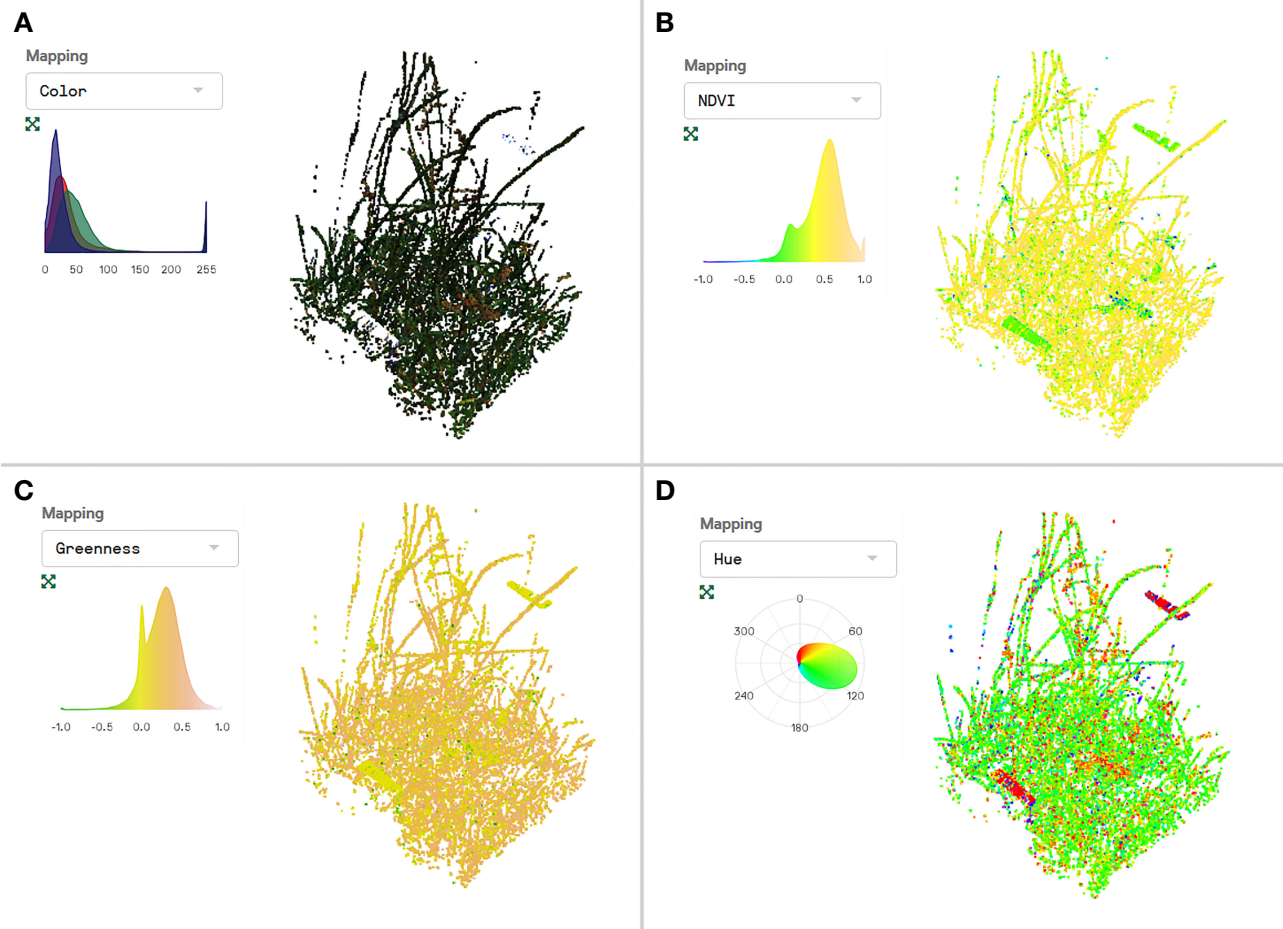
Visualization of the 3D point cloud of a single scan from digital whole-community phenotyping (DWCP) with the built-in software HortControl (Phenospex, Heerlen, The Netherlands). Each point within the cloud contains information on the position (X, Y, Z coordinates) as well as the reflection of red (620-645 nm), green (530-540 nm), blue (460-485 nm) and near-infrared (820-850 nm) wavelengths. Based on this information, RGB color **(A)**, NDVI **(B)**, greenness **(C)** and hue [°] **(D)** can be visualized (more options available in HortControl). The distribution of data is shown in the histograms **(A–C)** or in the color wheel where hues are arranged in a circle **(D)**. The sod originates from plot AEG 09 from the Exploratory Schwäbische Alb and the scan has been made in July 2020 (scan event 1, CW 26).

We used the automated plant phenotyping system PlantEye F500 (Phenospex, Heerlen, The Netherlands) to gather multispectral and structural information about plant communities. The PlantEye is equipped with an active sensor that projects a laser line with a wavelength of 940nm vertically onto the vegetation and records the reflection of the laser and the reflectance in Red, Green, Blue, and Near-Infrared with an integrated tilted camera (Kjaer and Ottosen, 2015). All 2D height profiles that were captured by moving the scanner over the plants, driven by an electric motor on a linear spindle axis, are then batched to generate a 3D point cloud. Each data point contains information on the 3D position in a coordinate system (X, Y, Z coordinates) as well as the reflection of red, green, blue, and near-infrared wavelengths (red = 620-645 nm, green = 530-540 nm, blue = 460-485 nm, near-infrared = 820-850 nm). Point clouds were processed with the built-in software HortControl (Phenospex, Heerlen, The Netherlands) that provides morphological and physiological parameters (**Table 1**). The software also visualizes the 3D point clouds allowing for a quick assessment of scans and the spatial distribution of structural and physiological parameters within the scans (**Figures 1A–D**). The PlantEye is independent from light conditions and sufficiently rain- and dustproof for outdoor use. The software processing the scans corrected the intensity of the reflected light for the distance between the vegetation and the sensor using the 3D data. In this study, each scan covered an area of 450 mm width and 300 mm length and 700 mm in height. Each scan had a resolution of <1 mm/pixel, comprising between 155,947 and 425,210 (mean ± sd = 263,692 ± 44,779.07) data points per scan.

**Table 1:** Parameters from digital whole-community phenotyping calculated by the software HortControl (for further information see: https://phenospex.helpdocs.com/plant-parameters/planteye-parameters).

Name	Description	Functional importance	Small values	High values	Potential range	Mean value	95% Cl	References
**Digital biomass [cm³]**	standing biomass; height*3D leaf area	proxy for plant productivity	low biomass	high biomass	0 - ∞	31,459,627	30,752,299 - 32,166,955	Quétier et al. (2007); Guo (2007)
**Height [mm]**	average plant height of the community; range from soil to the average top 10% of plants	proxy for plant productivity, nutrient availability, competition for light and growth strategy	lower (maximum) plant height	larger (maximum) plant height	0 - ∞	296.256	289.705 - 302.808	Falster and Westoby (2003); Quétier et al. (2007); Moles et al. (2009)
**Height max [mm]**	highest scanned point of the community; range from soil to highest point of plants				0 - ∞	538.317	532.661 - 543.973	
**Leaf area [cm²]**	3D leaf area; digital leaf area corrected for leaf inclination	proxy for heat load, water retention and gas exchange	small total leaf area	large total leaf area	0 – ∞	107,655.70	106,669.4 - 108,642.0	Stubbs and Wilson (2004)
**Leaf area index [cm²/cm²]**	leaf area per unit sector size; (Leaf Area)/(sector size)	related to photosynthetic activity, respiration and rainfall interception	community of low structural complexity	community of high structural complexity	0 – ∞	0.652	0.645 - 0.658	Fang et al. (2019); Knops and Reinhart (2000)
**Leaf area projected [cm²]**	2D leaf area; amount of sector size covered by leaves	proxy for plant development, growth and competition	sparse plant foliage	large area covered by leaves	0 - sector size	53,832.12	53,080.02 - 54,584.22	Lati et al. (2011)
**Leaf angle [°]**	average angle of leaves; average angle of the leaf surfaces to the perpendicular	proxy for light capture and water retention, indicator for plants’ strategies in dealing with abiotic factors	0° = vertical leaf	90° = horizontal leaf	0 - 90°	32.928	32.703 - 33.153	Craine et al. (2001); Stubbs and Wilson (2004)
**Leaf inclination [cm²/cm²]**	expresses how erected leaves are in average; (Total Leaf Area)/(Projected Leaf Area)		value of 1 = horizontal leaf	higher values = more vertical leaf	1 – ∞	1.880	1.868 - 1.891	
**Light penetration depth [mm]**	deepest point the laser can reach through the canopy; distance between the averaged bottom 20% and top 10% of vertical laser depth	measure for vegetation density and thus proxy for resource availability and above-ground competition	low vegetation density	high vegetation density	0 – ∞	192.251	187.447 - 197.055	Wilson and Tilman (1993); Quétier et al. (2007)
**NDVI (Normalized Digital Vegetation Index) [unit-less]**	ratio of reflected red light to near-infrared light; (NIR – RED)/(NIR + RED)	proxy for vegetation type and productivity, proxy for growth parameters like leaf area index and biomass	-1 – 0 = dead plants or non-plant objects, 0.1 – 0.2 = bare soil, 0.2 – 0.5 = unhealthy/sparse vegetation, > 0.5 = healthy/dense vegetation		-1 - 1	0.497	0.493 - 0.501	Myneni and Williams (1994); Senay and Elliott (2000); Thenkabail et al. (2000); Kaur et al. (2015)
**NPCI (Normalized Pigments Chlorophyll ratio Index) [unit-less]**	ratio of red and blue channel; (RED − BLUE)/(RED + BLUE)	associated to the chlorophyll content of a plant and thus proxy for the plants’ physiological state	lower red reflectance ≙ higher chlorophyll content (0.15-0.25 = green vegetation)	higher red reflectance ≙ lower chlorophyll content (>0.25 = stressed vegetation)	-1 - 1	0.152	0.150 - 0.155	Peñuelas et al. (1994); Bannari et al. (2007)
**PSRI (Plant Senescence Reflectance Index) [unit-less]**	ratio of red & green to near-infrared channel; (RED − GREEN)/(NIR)	associated to the carotenoid/chlorophyll content and thus proxy for leaf senescence	higher chlorophyll content (-0.1 to -0.2 = green vegetation)	lower chlorophyll content (> 0.2 = senescent vegetation)	-1 - 1	0.133	0.130 - 0.137	Merzlyak et al. (1999); Ren et al. (2017)
**Greenness/Green leaf index [unit-less]**	shows the relation of the reflectance in the green channel to the red & blue channel; (2*G-R-B)/(R+G+B)	proxy for combined effects of leaf physiology and canopy structure	more green vegetation	less green vegetation	-1 - 1	0.240	0.237 - 0.243	Keenan et al. (2014)
**Hue [°]**	the visible color dependent on the wavelength of light being reflected; expression of color taking into account all three measured colors (RGB) but independently from saturation and brightness value	proxy for the level of photosynthetic pigments and nutrient concentration in plant leaves	green vegetation ~120° (0°-60°=red, 60°-120°=yellow, 120°-180°=green, 180°-240°=cyan, 240°-300°=blue, 300°-360°=magenta)		0° - 360°	93.653	92.987 - 94.319	Adams et al. (1998); Widjaja Putra and Soni (2017)

**Figure 2:**
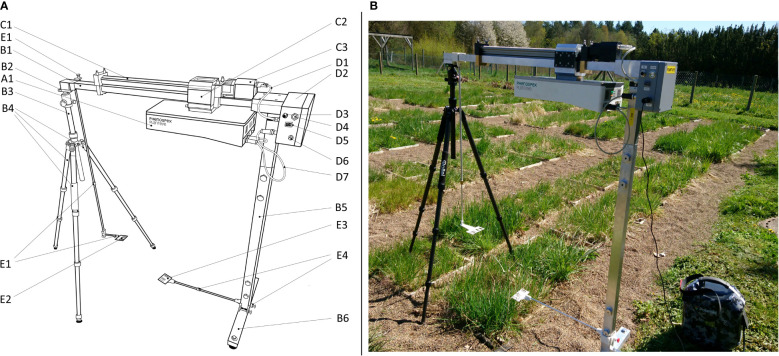
Customized mobile frame allowing the use of the PlantEye plant scanner in the field. **(A)** Construction plan of the customized plant scanner: A1) plant scanner, B1) horizontal bar, B2) mounting of the tripod, B3) carbon tripod, B4) length-adjustable legs, B5) vertical bar, B6) rotatable foot, C1) electric linear spindle axis, C2) slide, C3) motor, D1) motor power connection, D2) housing for electronic control unit, D3) port for computer connection, D4) start button, D5) speed control, D6) power cable connection, D7) scanner connection cables, E1) adjustable stop barcode bracket, E2) stop barcode, E3) start barcode, E4) adjustable start barcode bracket. Dimensions of the assembled frame: height = 126.7 cm, length = 122 cm and a width = 50.7 cm. **(B)** Photograph of the customized plant scanner for digital whole-community phenotyping in the field.

For mobile usage, the PlantEye can be mounted onto a mobile frame (MicroScan, Phenospex, Heerlen, The Netherlands) that we customized for use in the field (**Figures 2A, B**). We replaced the rear vertical bar with a lightweight carbon tripod (Rollei C6i, RCP Handels-GmbH & Co. KG, Germany), allowing for a much easier positioning on uneven ground while also minimizing the impact on the vegetation. The second vertical bar is replaced by a more robust and lightweight aluminum square tube. Such a tube also stabilizes the horizontal bar that contains the linear spindle axis, providing a better distribution of the weight of the scanner. A solid and waterproof housing contains the electronic control unit, where the horizontal speed of the scanner can be adjusted and scans can be started. An external portable battery was used as outdoor power supply (Polaroid PS600, Polaroid International B.V., 1013 AP Amsterdam, The Netherlands). The final re-built frame had a height of 126.7 cm, a length of 122 cm and a width of 50.7 cm at the stands (**Figure 2A**). A start and stop barcode, 3D printed with customized adjustable brackets to clip them to the square tubes, indicate the start and the end of the scanning area and also served as reference points to define the above-ground height (**Figure 2A**).

### Plant communities

To test the potential of digital phenotyping in plant and community ecology, we used plant communities arranged in a common garden established in the Botanical Garden of the University of Marburg, Germany. It was established in April to May 2020 and contains 156 sods. The grass sods originate from the three long-term research sites of the Biodiversity Exploratories (DFG Priority Programme 1374) located in the Biosphere Reserve Schwäbische Alb (ALB) in south-west Germany, the National Park Hainich (HAI) and its surroundings in the center and the Biosphere Reserve Schorfheide-Chorin (SCH) in the northeast of Germany (for further detail about the design see Fischer et al., 2010). We collected 13 sods from each region, which were selected to best represent a land-use gradient from all three regions and were then split into four parts of 50 x 50 cm. Each part was subsequently randomly assigned to one of four experimental land-use treatments. Sods that received the same treatment were arranged in one of three treatment blocks resulting in overall twelve blocks (further details about the set up can be found in the additional file).

Treatments differed in mowing frequency and application of fertilizer in a full factorial design: ‘00’ (= mowing once per year), ‘0M’ (= mowing twice per year), ‘F0’ (= mowing once per year and fertilizing once per year) and ‘FM’ (= mowing twice per year and fertilizing once per year). Land-use treatments started in mid-July 2020 where all sods were mown and sods assigned to ‘Fertilizer’ and ‘Mowing & Fertilizer’ treatments were fertilized. Mowing was performed using an electric grass cutter (Cordless Grass Shear DUM604RFX, Makita Werkzeug GmbH, Ratingen, Germany), which was used to cut the vegetation to about 2cm in height. The second mowing of sods assigned to ‘Mowing’ and ‘Mowing & Fertilizer’ treatments took place at the beginning of September 2020. In 2021, sods assigned to ‘Mowing’ and ‘Mowing & Fertilizer’ treatments were mown at the end of May and the fertilizing of sods assigned to the respective treatments was done right after. The second mowing in 2021 of all sods in the common garden took place in September. Per sod assigned to a fertilizer treatment, we applied 10.3 g fertilizer granulate (Yara Bela Sulfan, YARA GmbH & Co. KG, Dülmen, Germany), which corresponds to 99 kg N ha^-1^ year^-1^.

### Scanning and manual trait measurements

Over the course of 2020 and 2021, we scanned each plant community in the common garden eleven times (*n* = 11 scan events; 2020: 1) 23.-24.06, 2) 05.-06.08, 3) 26.-27.08, 4) 02.-03.09, 5) 22.-23.09, 6) 02.-03.11; 2021: 7) 26.-27.04, 8) 08.-09.06, 9) 07.-08.07, 10) 09.-10.08, 11) 08.-09.09) using the customized PlantEye scanner. For the scans we used the following software settings in HortControl: pot height = 0, start barcode Z = 170, stop barcode Z = 420, stop barcode y = 300, unit length = 300, length offset = 0, unit width = 450, width offset = -225, height start barcode = 150, height stop barcode = 400. The start barcode was positioned 15 cm above ground to minimize shadow casting on the vegetation and to increase the proportion of the vegetation covered by the plant scanner. The stop barcode was positioned 40 cm above ground to avoid shadow casting of vegetation on the barcode, which would lead to detection problems by the plant scanner. Scanning started five weeks after the common garden was established and was performed in a randomized order that differed in each of the scanning events in order to prevent a spatial and temporal bias in the data. Scan data were complemented with data on the taxonomic composition of the sods as well as with manual measurements of plant traits. Therefore, vegetation surveys were conducted in June/July 2020 and 2021 where all vascular plant species were identified and their cover was estimated with a resolution of 1%. In 2020 we recorded trait values for all plant species that covered >5% in a sod, including plant height [cm], leaf length [cm] and width [cm], leaf dry weight [g] and specific leaf area [mm^2^ mg^-1^] following the protocol of Junker et al. (Junker and Larue-Kontić, 2018; Junker et al., 2020). Plant height was measured in the field to the nearest 1 mm using a folding rule. We collected up to five leaves from one to three individuals per species and sod to determine the respective leaf area by scanning the leaf using an office scanner (Perfection 2400 Photo scanner, Seiko Epson Corporation, Nagano, Japan). Leaf area was calculated from the digital leaf scans by dividing the number of pixels per leaf by the number of pixels of a reference square centimeter as obtained from the GNU Image Manipulation Program (GIMP), version 2.10.20 (The GIMP Development Team 2020, retrieved from https://www.gimp.org). After scanning, leaves were dried for at least two days at 60°C and weighted on a precision scale (Kern ABS80-4N, KERN & SOHN GmbH, Balingen. Germany). The specific leaf area (SLA) was then calculated by dividing leaf area by leaf dry weight. Additionally, total plant biomass of each grass sod was dried separately after mowing for at least four days at 60°C, then subsequently weighted (Sartorius L 610-D, Sartorius AG, Göttingen, Germany) for a direct measure of dry weight biomass [g].

### Statistical analyses

All statistical analyses were conducted in R (R Core Team, 2022). To test the validity of PlantEye parameters, we compared the digital biomass resulting from digital whole-community phenotyping with the biomass weighted after removing it from the sods shortly after the scans.

To test the explanatory power of DWCP, the plant species composition and the community weighted means (CWM) of manual trait measurements, we ran multiple classifications using *“random forest”*, a machine learning algorithm that assigns samples, in this case the 156 sods in the common garden, to predefined groups: provenance (3 Exploratories), land use treatments (4 experimental land-use treatments), mowing (Y/N) and fertilizer (Y/N) in multiple iterations and estimates the importance of each parameter to achieve the best possible classification (Breiman, 2001). We used three sets of explanatory variables to predict provenance, treatment, mowing and fertilizing: 1) 14 parameters from DWCP (parameters provided by HortControl) with scan events as subsets. 2) plant species list with estimated cover for 2020 and 2021. 3) community weighted mean (CWM) of leaf length, leaf width, leaf dry mass, leaf area and specific leaf area, calculated with the R package *FD* (Laliberté and Legendre, 2010) with species-specific trait values and the species abundances from 2020 and 2021, respectively. All random forest classifications were run with the R package *randomForest* (Liaw and Wiener, 2002) with *mtry* = 4 variables randomly sampled at each split of a decision tree and *ntree* = 10,000 trees to grow for all classifications. We used accuracy values calculated from *randomForest* confusion matrices with the R package *caret* (Kuhn, 2022, version 6.0-86) to quantify classification performance. With the function ‘importance’ of the R package *randomForest*, we extracted variable importance to identify the top five variables that improved classification (Cutler et al., 2007). Note that many other PlantEye parameters differed among treatments as well (**Table 2**).

**Table 2:** ANOVA and t-test results of differences between Exploratories (sod origin), land-use treatments, mowing treatment (Y/N) and fertilizer treatment (Y/N) for all 14 parameters from digital whole-community phenotyping.

	Sod origin		Land-use treatment		Mowing treatment		Fertilizer treatment	
	F_2,152_	p-value	F_3,148_	p-value	*t*_150_	p-value	*t*_152_	p-value
Digital biomass	21.25	**<0.001**	221.3	**<0.001**	23.436	**<0.001**	-7.8332	**<0.001**
Height	16.12	**<0.001**	338.7	**<0.001**	30.265	**<0.001**	-3.2082	**0.002**
Height max	26.16	**<0.001**	383.4	**<0.001**	34.098	**<0.001**	-1.7917	0.07517
Leaf area	10.64	**<0.001**	6.531	**<0.001**	3.0027	**<0.001**	-9.4282	**<0.001**
Leaf area index	10.63	**<0.001**	6.524	**<0.001**	3.0032	**<0.001**	-9.4243	**<0.001**
Leaf area projected	12.09	**<0.001**	7.792	**<0.001**	2.644	**<0.001**	-8.8662	**<0.001**
Leaf angle	10.87	**<0.001**	11.62	**<0.001**	3.4463	**<0.001**	-3.413	**<0.001**
Leaf inclination	10.38	**<0.001**	11.19	**<0.001**	-3.3461	**<0.001**	3.2513	**<0.001**
Light penetration depth	17.14	**<0.001**	228.9	**<0.001**	25.659	**<0.001**	-0.80226	0.4237
NDVI	4.174	**0.017**	228.9	**<0.001**	6.2647	**<0.001**	-10.067	**<0.001**
NPCI	22.41	**<0.001**	104.5	**<0.001**	-7.9648	**<0.001**	10.813	**<0.001**
PSRI	2.287	0.105	42.18	**<0.001**	-7.2645	**<0.001**	9.9185	**<0.001**
Greenness	4.659	**0.011**	15.37	**<0.001**	5.4095	**<0.001**	9.9185	**<0.001**
Hue	16.9	**<0.001**	66.11	**<0.001**	8.6115	**<0.001**	-9.0425	**<0.001**

Bold values indicate significance.

## Results and discussion

In total we analyzed *n* = 1679 digital whole-community phenotyping (DWCP) scans of *n* = 156 sods containing *n* = 167 plant species using the customized PlantEye scanner in summer 2020 and 2021. The mean values of the non-invasively measured community-wide parameters obtained by DWCP seem to give a valid representation of the actual plant community as indicated by the highly significant positive relationship between the weighted plant biomass removed from sods and the digital biomass extracted from scans (quadratic polynomial regression, p < 2.2e-16, adjusted R^2 = ^0.59, see Additional **Figure 3**), supporting results from scans of plant individuals (Laxman et al., 2018). The non-linearity of the relationship in the whole-community scanning approach and the wider scattering with increasing total biomass can primarily be explained by two factors. First, the laser is not able to penetrate foliage, which leads to a higher error for denser vegetation. Second, digital biomass is calculated using height and 3D leaf area (see **Table 1**) and thus also sensitive to large overlap of plants within a scan.

**Figure 3:**
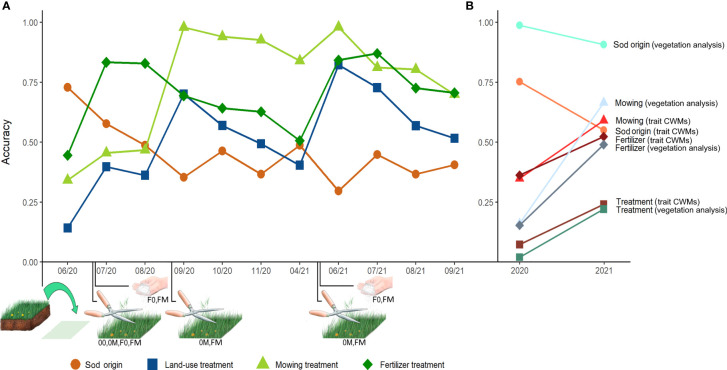
**(A)** Accuracy of random forest classifications (ntree = 10,000; mtry = 4) of plant communities (= sods) to sod origin (circle, 3 Exploratories), treatments (square, 4 experimental land-use treatments*), mowing (triangle, Y/N) and fertilizer (rhombus, Y/N) over the course of 2020 and 2021 with data from digital whole-community phenotyping. Time points of common garden establishment and mowing and fertilizer application events are indicated along the x-axis, treatments affected by these events are indicated below illustrations. **(B)** Accuracy of random forest classifications with data from vegetation analysis and community weighted means (CWMs) of traits. As vegetation analyses have been performed only once per year, only changes in accuracy between two years are displayed. *’00’= mowing once per year; ‘0M’= mowing twice per year; ‘F0’= mowing once per year and fertilizing once per year; ‘FM’= mowing twice per year and fertilizing once per year.

We used the machine learning algorithm random forest to assign sods to either sod origin (three regions of the Biodiversity Exploratories), experimental land-use treatment (four treatments), mowing treatment (mown once or twice), or fertilizer treatment (with or without fertilizer addition). Note that the probability of a correct classification by chance differs between these analyses due to different numbers of categories. Classifications were performed based on the 14 parameters representing a mean value of each parameter across the whole plant community returned by the software HortControl. These mean values of morphological and physiological parameters can therefore be seen as an analog to community weighted means of manually measured traits. For a comparison of information content about changes in our plant communities, species composition and community weighted means (CWMs) of plant species’ traits were also used as explanatory variables for classification. Since CWMs were calculated from trait values recorded once in 2020 and species’ abundance of 2020 and 2021, respectively, they are only affected by changes in species composition and abundance, not by intraspecific trait-variation across years. While this certainly is a valid approach depending on the research question, here it also facilitates comparability between methods since DWCP cannot directly detect intraspecific changes in response to treatments.

**Figure 4:**
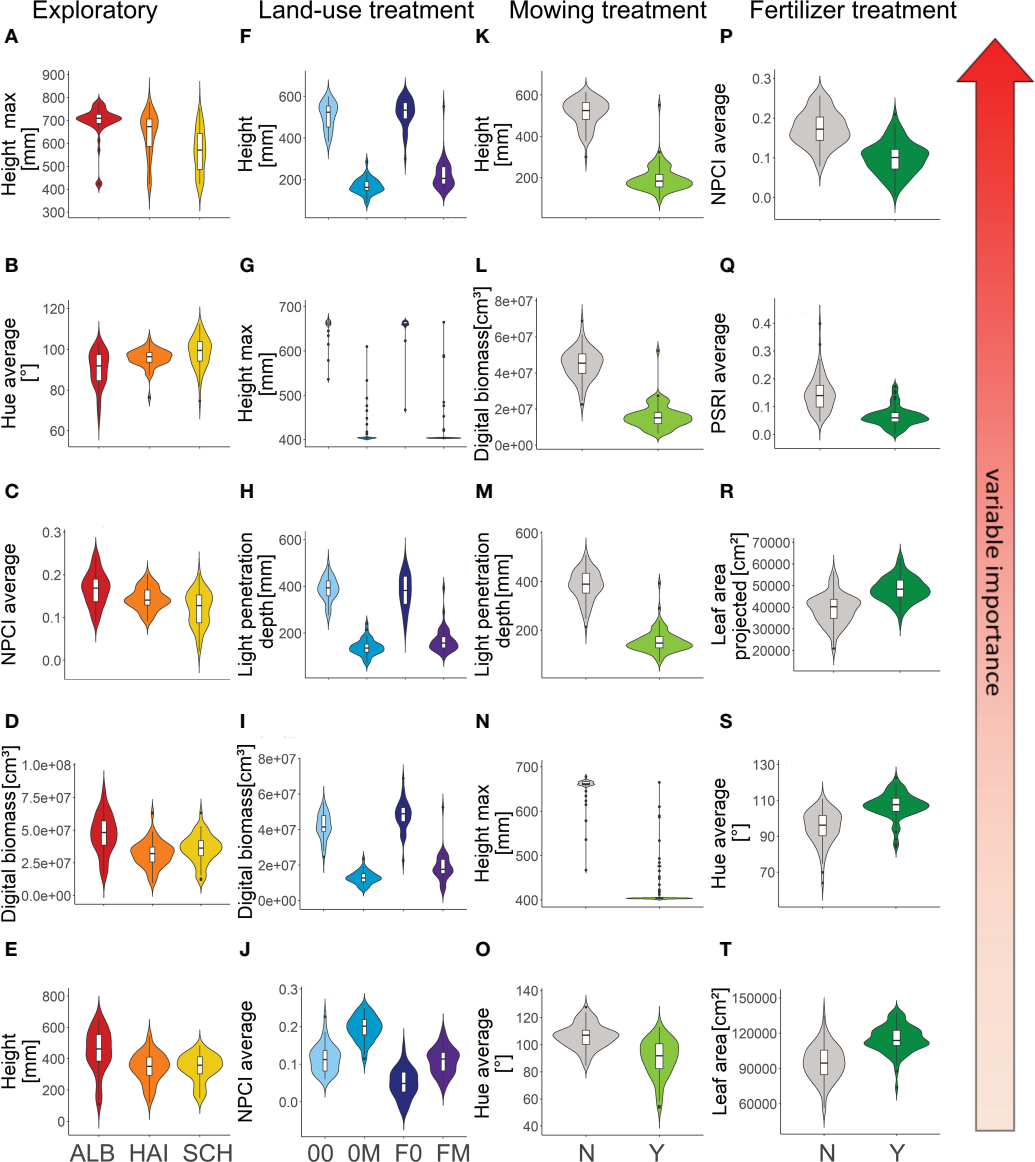
Differences between sod origin [**(A–E)** 3 Exploratories: ALB = Schwäbische Alb, HAI = Hainich, SCH = Schorfheide-Chorin], land-use treatments [**(F–J)** 0 = mowing once per year, 0M = mowing twice per year, F0 = mowing once and fertilizing once per year, FM = mowing twice and fertilizing once per year], mowing [**(K–O)** Y/N] and fertilizer [**(P–T)** Y/N] in five variables with the highest variable importance for classification in a descending order. For each dependent variable, results of the scan event that received the highest accuracy are shown (scan events 1, 8, 8 and 9, respectively). Boxplots display the distribution of the variables and also show the median, lower and upper quartiles, and outliers. The violin plot outlines depict density distribution of data. All supporting ANOVAs **(A–J)** and t-tests **(K–T)** were highly significant (for detailed ANOVA and t-test results see **Table 2**).

Accuracy of classification of sods to their origin (three regions of the Biodiversity Exploratories) by DWCP was highest shortly after the establishment of the common garden but strongly decreased in the following weeks and was on average lower throughout the rest of 2020 and the whole season of 2021 (**Figure 3A**). In contrast, species composition was strongly indicative for sod origin, resulting in a high classification accuracy in both 2020 and 2021 (**Figure 3B**). A classification based on CWMs of plant species’ traits in the sods was also successful in both years but with a reduced accuracy in 2021 (**Figure 3B**). The three regions of the Biodiversity Exploratories differ in plant species composition (Socher et al., 2013), prevalent soil types, climatic conditions and land-use history but cover a similar range of land-use intensities (Fischer et al., 2010; Klaus et al., 2013; Gilhaus et al., 2017). The fast decrease in accuracy of classification based on DWCP indicates that plant communities show fast responses in morphological and physiological properties to land-use treatments (see below) independent of land-use history, soil type and species composition. We could also observe changes in the relative abundance of plant species within communities, which resulted in temporal variability of CWMs within plots. In contrast, changes in plant species composition usually emerge only a few years after land-use changes (Read et al., 2018; Komatsu et al., 2019) and thus remained largely stable in our experiment. Accordingly, CWMs of species-specific traits reflect the species composition and thus also slowly responded to land-use changes. The relatively high accuracy in classification by DWCP shortly after the establishment of the common garden may reflect the climatic and edaphic differences in the three regions of origin that may result in differences in vegetation height, digital biomass, hue and NDVI (**Figures 4A–E**). In fact, it has been shown that climate and edaphic conditions affect plant productivity (Burke et al., 1998; Lobell et al., 2002; Hsu et al., 2012). Likewise, remote sensing studies demonstrated changes in NDVI (Tucker and Sellers, 1986; Paruelo et al., 1997) and hue (Dreesen et al., 2013) based on these factors. Additionally, the high accuracy of classification to sod origin based on DWCP may also be explained by the different arrival times of sods at the common garden. Sods from the three regions were transported to the common garden between April and May 2020 and thus the sods from different regions recovered for a different period of time (11 weeks for sods from Schwäbische Alb, 8 weeks for the sods from Hainich, 5 weeks for the sods from Schorfheide-Chorin) from transportation prior to the first scan, which may have resulted in differences in vegetation parameters as well.

Accuracies of classifications of sods to the four land-use treatments right after the establishment of the common garden were low for DWCP as well as species composition and CWMs of traits since the land-use treatments had not yet been conducted (**Figures 3A, B**). High peaks in accuracy directly following land-use treatments reveal a clear signal of these treatments detected by DWCP. By the end of the second year the accuracy of classification of sods to the land-use treatments by DWCP parameters has increased considerably, also in scans not directly following the land-use treatments (**Figure 3A**). As stated above, species composition of plant communities can be resistant to land-use change for some years (Read et al., 2018; Komatsu et al., 2019), which is also reflected in the still low, though slightly higher, classification accuracy of treatments from species composition and trait CWMs in 2021. (**Figure 3B**). In contrast to DWCP that allows assessments in high frequencies, vegetation analysis was performed only once per year and trait measurements only once per species. Thus, differences in classification accuracy may also be attributed to differences in the temporal resolution of data collection revealing a clear advantage of DWCP compared to classical methods. In addition, DWCP measures various morphological and physiological parameters, thus providing a multifaceted view on plant communities that are indicative for different land-use treatments (**Figure 4**). In remote sensing studies it has been shown that multiple indices outperform single indices in assessing the properties of plant communities (Filella et al., 1995; Hollberg and Schellberg, 2017), which is confirmed by our results. Land-use treatments mostly affected vegetation height, light penetration depth and digital biomass (**Figures 4F–I**), reflecting the strong changes in standing biomass by mowing or fertilizer addition that promotes plant growth and above-ground biomass (Quétier et al., 2007). Note that the parameter ‘maximum height’ is associated with some limitations as it is limited by the maximum height the scanner is able to capture, but discriminates mown from unmown plots with a high accuracy. NPCI as a proxy for the proportion of total photosynthetic pigments to chlorophyll (Peñuelas et al., 1994; Bannari et al., 2007; Hollberg and Schellberg, 2017) was also indicative for land-use treatments (**Figure 4J**). Higher nitrogen availability after fertilizer addition leads to higher chlorophyll concentrations in leaves and consequently to lower NPCI values (Kantety et al., 1996; Schlemmer et al., 2013). In contrast, mowing exposes previously shaded plant parts with lower chlorophyll concentrations (Madison, 1962; Wang et al., 2018) and thus leads to higher NPCI values (**Figure 4J**).

Classification of sods separately to mowing and fertilizer treatments shortly after the establishment of the common garden and prior to the application of the treatments was, as expected, not successful for DWCP, species composition, and CWMs of plant traits (**Figures 3A, B**). In both years, the accuracy of classification with DWCP peaked shortly after the respective treatment was conducted, except for the DWCP measurements after the first mowing treatment in 2020 where all sods have been mown. Mowing clearly resulted in structural differences in the plant communities like vegetation height, digital biomass and light penetration depth (**Figures 4K–N**). Strong differences in hue between mown and unmown sods (**Figure 4O**) can be explained by the exposure of previously shaded plant parts with lower chlorophyll concentrations (Madison, 1962; Guertal and Evans, 2006; Wang et al., 2018) and by increased leaf senescence subsequently leading to chlorophyll loss at the cut surface (Howieson, 2001). Therefore, mown sods shifted from green to more yellow colors (**Figure 4O**). In fact, all 14 parameters differed significantly between mown and unmown plant communities (see **Table 2**). Fertilizer treatment was mainly indicated by physiological parameters (**Figures 4P–T**). Fertilizer leads to a higher nitrogen and chlorophyll content in leaves which is measured by both NPCI and PSRI (España-Boquera et al., 2006; Bannari et al., 2007; Dong et al., 2012). NPCI is negatively related to chlorophyll content and thus unfertilized sods had higher values than fertilized sods (**Figure 4P**). PSRI increases with degree of senescence due to the resulting loss of chlorophyll and accumulation of carotenoids in leaves (Merzlyak et al., 1999). N fertilizer reduces leaf senescence and promotes higher chlorophyll levels (Wolfe et al., 1988; Dong et al., 2012), both leading to lower PSRI values in fertilized plants (**Figure 4Q**). Hue was slightly higher in fertilized sods (**Figure 4S**), indicating overall stronger, more intense green color of plants after the application of fertilizer, most likely caused by a higher chlorophyll concentration (Adams et al., 1998; Widjaja Putra and Soni, 2017). The higher 2D and 3D leaf area of fertilized sods (**Figures 4R–T**) may be caused by an increase in vegetation density as well as cover density (Quétier et al., 2007) and increased leaf areas (Evans, 1983) after fertilizer treatments. Indeed, all parameters but maximum height and light penetration depth were significantly different in fertilized compared to unfertilized plots (see **Table 2**). In the first season (2020) after the establishment of the common garden, classification of mowing treatments was more accurate than classification of fertilizer treatments. In the second season (2021), accuracies for both treatments were similarly high (**Figure 3A**), suggesting that the effects of fertilizer treatments that accumulate in plant communities are permanently visible only after a second application. Classification accuracies of sods to mowing or fertilizer treatment based on species composition and CWMs of traits increased slightly from 2020 to 2021 but remained lower than classification by DWCP (**Figures 3A, B**). As mentioned above, plant community composition can be resilient to land-use changes for a period of time (Read et al., 2018; Komatsu et al., 2019) albeit clear differences in species composition due to long-term differences in land-use (Socher et al., 2013). Therefore, short-term responses in vegetation properties as detected by DWCP and measurable in a high temporal resolution may be an indicator for future shifts in species composition and ecosystem properties.

## Future directions

Our data show that digital whole-community phenotyping (DWCP) detects plant community responses to changes in abiotic parameters much earlier than these changes become evident in changes in plant traits, species composition or abundance. Plant traits have been shown to be valuable indicators for ecosystem states (Quétier et al., 2007; Vandewalle et al., 2010) and for tipping points often associated with irreversible changes in ecosystems (Dakos et al., 2019). Likewise, remote sensing data have been utilized to identify grassland land-use intensity focusing on spectral information of pixels (Gómez Giménez et al., 2017). DWCP may bridge the gap between trait measurements in the field and remote sensing and provides a fast and precise method to infer land-use intensity from a combination of morphological and physiological data. Furthermore, the ability to detect community-wide responses to changes in land-use intensity in advance to alterations of species composition and irreversible changes in ecosystems may allow sufficient time to take countermeasures to conserve grasslands.

Future software developments may further increase the potential of DWCP in quantifying community or plant species traits and also as proxies for plant species diversity in communities. The software HortControl, designed to analyze the scans of plant individuals, integrates a whole community and returns community weighted means of the parameters listed in **Table 1**. Using raw data from digital phenotyping devices, machine learning algorithms succeeded in the segmentation of plant individuals, meaning that morphological structures are recognized in point clouds (Ghahremani et al., 2021; Turgut et al., 2022). In dense grassland plots, this task may be more challenging as individual plants may overlap with other plants and plant parts are not that well separated as in scans of individual plants. Nonetheless, the seven-dimensional raw data (x, y, z position of each point in the point cloud and the red, green, blue, and near-infrared channel) represent a playground for future developments for their analyses and provide the potential for deeper insights into the properties of plant communities.

## Conclusion

Digital whole-community phenotyping turned out to be an efficient method to measure morphological and physiological characteristics of plant communities and thus complements other trait-based approaches and may bridge the gap to remote sensing. Our data suggest that community-wide responses to abiotic parameters are independent on plant species composition and diversity and therefore DWCP represents the next level of generalization attributed to trait-based approaches. Future scans in other ecosystems are, however, required for an assessment of the similarities and differences in plant community responses to land-use changes or other abiotic influences. We conclude that bringing digital plant phenotyping from the lab or greenhouse into the field will reveal detailed insights into the morphological, physiological and functional responses of plant communities in a relevant ecological context.

## Data availability statement

The datasets presented in this study can be found in online repositories. The names of the repository/repositories and accession number(s) can be found below: http://doi.org/10.17616/R32P9Q.

## Author contributions

RJ conceived the study. VZ and RJ designed the study. VZ performed field work. VZ and RJ performed statistical analysis. VZ drafted the first version of the manuscript. All authors contributed to the article and approved the submitted version.
