# Supplementary material for: Digital whole-community phenotyping: tracking morphological and physiological responses of plant communities to environmental changes in the field
Source: Front Plant Sci. 2023 May 9;14:1141554. doi: 10.3389/fpls.2023.1141554 (PMC10203609; doi:10.3389/fpls.2023.1141554)
Supplement: Supplementary file 1 [file DataSheet_1.pdf]

Supplementary Material

Supplementary Data: Further details on the common garden 'EXClAvE', the experimental land-use treatments, preparations prior to the establishment of the common garden and field work specifics.

The Biodiversity Exploratories are a large-scale research network where the land-use and biodiversity of 50 grassland plots per region have been continuously recorded since 2006 (Fischer et al., 2010). From each of the three regions ('Exploratories') Schwäbische Alb, Hainich and Schorfheide-Chorin, we selected 13 plots that evenly cover the range of land-use intensity (measured as land-use index LUI, see Blüthgen et al., 2012). In each of the 39 plots, we collected grass sods of the size of 1m2 and 10cm thickness in April and May 2020. Every sod was split into four parts of 50x50cm to cover all four experimental land-use treatments and then allocated to one of three treatment blocks that consisted of 14 sods that will be receiving the same experimental land-use treatment: 13 sods from the Exploratories plus one square with steam-sterilized soil to monitor spontaneous plant establishment from the surroundings. Since we have four treatments divided into three blocks each, in total, the common garden consists of twelve treatment blocks (Additional file 2), adding up to 168 sods. Sods of fertilizer treatments were treated with 99 kg Nitrogen per ha per year, which corresponds to 10.3 g per sod of the industrial fertilizer 'YaraBela Sulfan' (YARA GmbH & Co. KG, Dülmen, Germany), a 24% nitrogen multi-nutrient fertilizer widely used on grassland areas within the Biodiversity Exploratories framework. The fertilizer granulate was evenly distributed on each sod per hand. The amount of fertilizer was calculated from the 90% quantiles of land-use management intensity on the grassland plots of the Biodiversity Exploratories, based on the mean fertilization, mowing and grazing in the years 2006 to 2016, which has also been used to determine the number of cuts per year. Before the arrival of the grass sods in Marburg, roughly 14 cm of soil were removed from the area of the common garden and subsequently filled again with about 4 cm of gravel to impede upwards root growing. We used wooden frames built from untreated wood with the internal dimension of 50x50 cm as boundaries for the grass sods. After placing the wooden frames, we collected the grass sods from the Schwäbische Alb, National Park Hainich and Schorfheide-Chorin over the course of April 2020 and always planted the sods a maximum of 4 days after collection. In the field, grass sods the size of one square meter were excavated with spades. We then divided them into four equally sized sods (0.25 m2) and transported them to Marburg in plastic boxes. The already harvested sods have been watered during storage until they were planted into the wooden frames in the common garden. During the initial post-planting period sods were sufficiently watered to ensure proper setting of the plants and a good rooting. After the successful planting of all 168 sods, areas between sods were covered with anti-weed fabric (PPX 100g/m2 Bändchengewebe, Hermann Meyer KG, Rellingen, Germany) before being filled with gravel to the height of the wooden frames. When necessary for survival, grass sods were additionally watered evenly over the whole area of the common garden using two sprinklers (Gardena AquaZoom M, GARDENA GmbH, Ulm, Germany).

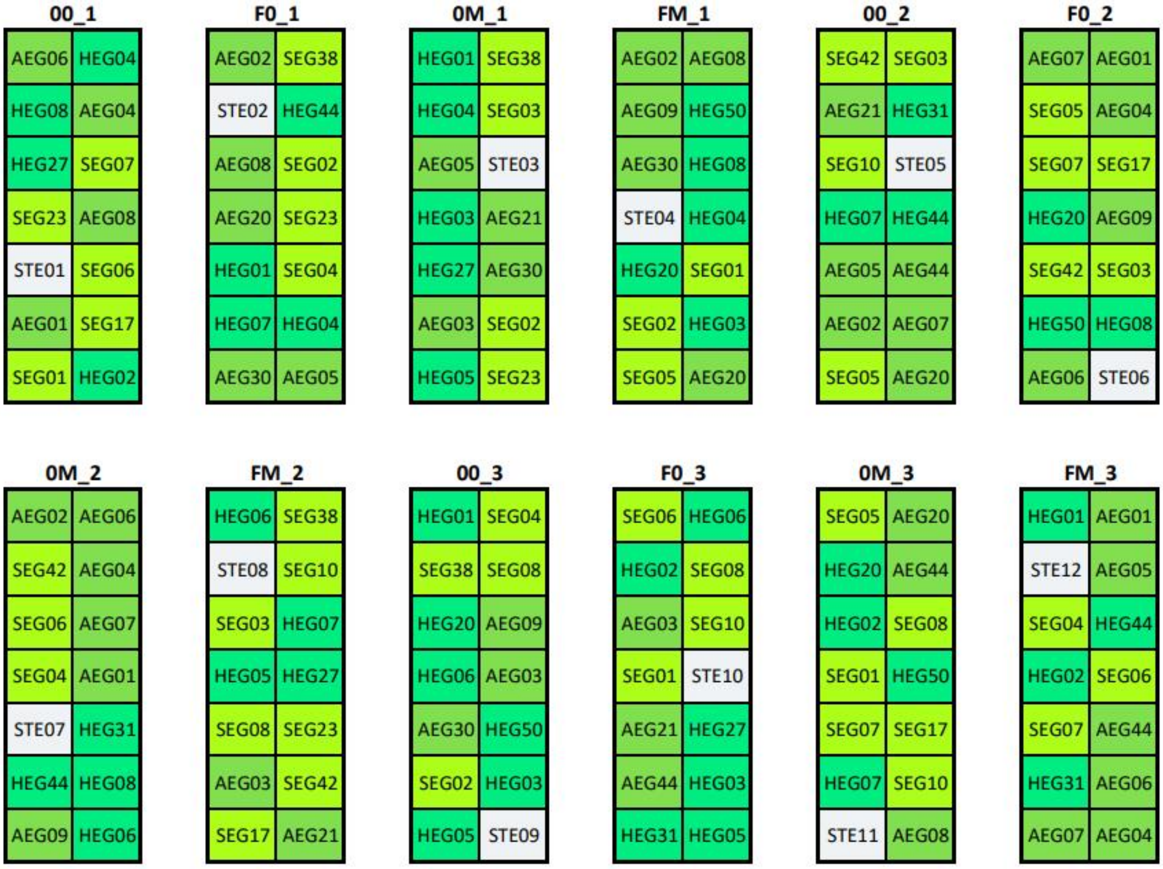

Supplementary Figure 1: Setup of plant communities in the common garden at the Botanical Garden of the University of Marburg, Germany. Established April to May 2020, 156 sods originate from the three research sites of the Biodiversity Exploratories (Biosphere Reserve Schwäbische Alb (ALB), National Park Hainich (HAI) and its surroundings, Biosphere Reserve Schorfheide-Chorin (SCH)). Sods were split into four parts of 50 x 50 cm and each part was randomly assigned to one of four experimental land-use treatments (0 = mowing once per year, 0M = mowing twice per year, F0 = mowing once and fertilizing once per year, FM = mowing twice and fertilizing once per year). Sods that received the same treatment were arranged in one of three treatment blocks resulting in overall twelve blocks.

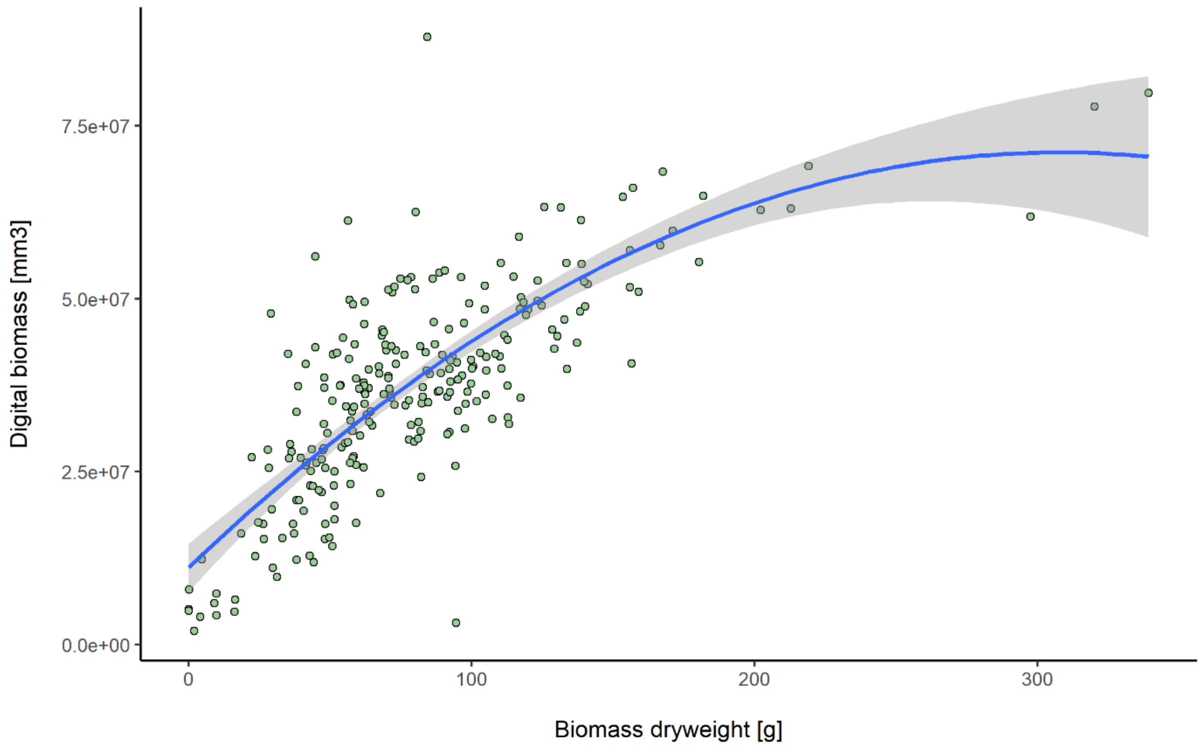

Supplementary Figure 2: Quadratic polynomial regression between digital biomass as extracted from scans and the weighted plant biomass removed from sods. The fitted model yielded highly significant p-values for both linear and quadratic term ($p < 2e\text{-}16$ and $p = 1.10e\text{-}07$, respectively). The resulting p-value $p < 2.2e\text{-}16$ and adjusted R-squared $R2 = 0.59$ suggest a highly significant positive relationship, confirming that parameters from digital whole-community phenotyping (DWCP) and manual (invasive) measurements are comparable. The non-linearity of the relationship that is noticeable by general variation and increasing scattering with higher total biomass primarily results from the fact that the laser is not able to penetrate foliage, thereby leading to a higher error for denser vegetation, and also from the formula to calculate digital biomass (product of height and 3D leaf area, see Table 1).
